# Physical activity and calorie intake mediate the relationship from depression to body fat mass among female Mexican health workers

**DOI:** 10.1186/s12966-017-0612-x

**Published:** 2017-11-17

**Authors:** Amado D. Quezada, Nayeli Macías-Waldman, Jorge Salmerón, Tessa Swigart, Katia Gallegos-Carrillo

**Affiliations:** 1 INSP. Center for Evaluation and Surveys Research, National Institute of Public Health. INSP, Cuernavaca, Morelos Mexico; 2 Center for Nutrition and Health Research, National Institute of Public Health of Mexico, Cuernavaca, Morelos Mexico; 3 Academic Unit in Epidemiological Research, Research Center in Policies, Population and Health, School of Medicine, National Autonomous University of Mexico, Ciudad de México, Mexico; 4 Center for Population Health Research, National Institute of Public Health, Cuernavaca, Morelos Mexico; 5 Unidad de Investigación Epidemiológica y en Servicios de Salud, Instituto Mexicano del Seguro Social, Boulevard Benito Juárez No. 31. Centro. C.P., 62000 Cuernavaca, Morelos Mexico

**Keywords:** Depression, Physical activity, Women, Mexico

## Abstract

**Background:**

Depression is a foremost cause of morbidity throughout the world and the prevalence of depression in women is about twice as high as men. Additionally, overweight and obesity are major global health concerns. We explored the relationship between depression and body fat, and the role of physical activity and diet as mediators of this relationship in a sample of 456 adult female Mexican health workers.

**Method:**

Longitudinal and cross-sectional analyses using data from adult women of the Health Workers Cohort Study (HWCS) Measures of body fat mass (kg from DEXA), dietary intake (kcal from FFQ), leisure time activity (METs/wk) and depression (CES-D) were determined in two waves (2004–2006 and 2010–2011). We explored the interrelation between body fat, diet, leisure time, physical activity, and depression using a cross-lagged effects model fitted to longitudinal data. We also fitted a structural equations model to cross-sectional data with body fat as the main outcome, and dietary intake and physical activity from leisure time as mediators between depression and body fat.

**Results:**

Baseline depression was significantly related to higher depression, higher calorie intake, and lower leisure time physical activity at follow-up. From our cross-sectional model, each standard deviation increase in the depression score was associated with an average increase of 751 ± 259 g (± standard error) in body fat through the mediating effects of calorie intake and physical activity.

**Conclusions:**

The results of this study show how depression may influence energy imbalance between calories consumed and calories expended, resulting in higher body fat among those with a greater depression score. Evaluating the role of mental conditions like depression in dietary and physical activity behaviors should be positioned as a key research goal for better designed and targeted public health interventions.

**Trial registration:**

The HealthWorkers Cohort Study (HWCS) has been approved by the Institutional IRB. Number: 2005–785-012.

**Electronic supplementary material:**

The online version of this article (10.1186/s12966-017-0612-x) contains supplementary material, which is available to authorized users.

## Background

Depression is a general word for altered, abnormal low psychological state that affects the thoughts, actions, and sense of well-being in an individual [1]. Depression can be a disabling condition that adversely affects individuals and relationships with their family and friends, work, sleeping and eating, and general health [2]. As the severity of depression increases, so does the risk of all-cause mortality, including deaths from strokes and cardiovascular events [3].

Depression is a foremost cause of morbidity throughout the world, and in 2010 Major Depressive Disorder (MDD) accounted for 2.5% of Disability-Adjusted Life Years lost worldwide [4]. The prevalence of depression in women is about twice as high as men, possibly due more to psychosocial factors than genetic or hormonal factors [5]. A study conducted in 2007, using a nationally representative sample of urban Mexican populations aged 18–65 years found an estimate of 26.1% of Mexicans who had experienced at least one psychiatric disorder in their life. The prevalence of any mood disorders was found to be 9.2% [6]. A different 2005 study by Bello et al., using data from the 2002–2003 National Assessment Performance Survey in Mexico, found the prevalence of depression to be 4.5% overall, with 5.8% among women and 2.5% among men [7]. Although Mexico does not have high rates of depression compared to other countries, it still accounts for a considerable amount of morbidity. Moreover, depression prevalence is most likely underestimated [7].

Additionally, overweight and obesity are major global health concerns given their rapid increase and their related health risks; from 1980 to 2013, the combined prevalence of overweight and obesity (BMI ≥ 25) increased from 28.8% to 36.9% in men and from 29.8% to 38.0% in women worldwide [8]. According to the National Health Survey in Mexico, the combined prevalence of overweight and obesity in Mexican women doubled from 34.5% in 1988 to 70.5% in 2012, and was found to be 3.6 percentage points higher in women compared to men [9].

Diet is also an important factor in both body fat and depression [10]. There is much evidence that the quality and quantity of one’s diet has a causal relationship in levels of adiposity, with greater intake of highly processed foods that are calorie-rich and low in fiber contributing more significantly to both BMI and body fat [11]. The relationship between diet and depression may be considered complex, but in general depression has a positive association with lower quality dietary habits [12, 13]. An important challenge is finding causality between depression, calorie intake, physical activity (PA), and body fat. The relationship between depression and fat is multifactorial, and the way these variables are interrelated may include reciprocity. Causality requires not only association but also directionality and isolation from common causes as well [14]. Experimental and longitudinal studies may prove to be relevant to elucidate these complex interrelations.

A meta-analysis that included 19 prospective studies showed that the relationship from depression to BMI was much stronger than in the opposite direction [15]. Regarding PA and depression, studies have shown an inverse association [16–18] and a positive association between sedentariness and depression [19]. The relationship between depression and BMI and their proposed mechanisms have been explored in cross-sectional studies using Structural Equations Models (SEM); in a study applied to NHANES data from 1999 to 2004, PA was found to mediate the relationship between depression and BMI [20]. Among Canadian women, another study found a significant relationship from depression to BMI but not in the opposite direction [21]. Although there are studies that have focused on the relationship between depression and BMI, there is a lack of evidence concerning the association between depression and body fat in general. BMI has been used as a proxy indicator for adiposity; however, BMI has a limited ability to distinguish body components. Furthermore, the use of modern imaging methods such as DEXA has made evident the necessity to use predictive indicators of body composition and clinical outcomes in a more accurate way. These methods allow researchers and clinician to improve the understanding of the relationship between body fat and health outcomes like depression [22].

Given the burden of both depression [7] and overweight/obesity in Mexico [8], investigation into the implied mechanisms would provide better insight into tackling the epidemic. Therefore, we explored the interrelation between body fat, diet, PA and depression in a sample of female Mexican health workers using both longitudinal and cross-sectional data with a SEM approach. We also looked at the role of PA and diet as mediators between depression and body fat. To the best of our knowledge this is the first study exploring these complex relationships in a Mexican population of adult woman and with body fat instead of indirect measures based on body weight and height.

## Methods

### Data

We conducted longitudinal and cross-sectional analyses using data from adult women of The Health Workers Cohort Study (HWCS). The HWCS is a follow-up study to determine the relationships between lifestyle factors and health in a Mexican population. The information about the study design and methodology has been previously published [23]. The participants were recruited using flyers and letters sent to the health workers, who were also asked to invite their relatives to take part in the study. 10,769 subjects were invited to participate in the HWCS of which 40 did not attend the medical examinations and blood sample collection; 10,729 subjects were included in the HWCS baseline measurement (2004–2006). In the follow-up during 2010–2011, 2500 were invited to participate and 1855 attended, with a response rate of 74.2%. The sample analyzed for this study comprised 456 female subjects with complete measurements at baseline (measurements taken from 2004 to 2006) and follow-up (from 2010 to 2011).

### Measurements

#### Depression

The symptoms of depression were measured with the self-administered scale version of the CES-D [24], which has been applied in several populations, including Spanish-speaking groups [25–27]. The original instrument consists of a 20-item questionnaire used to generate a continuous type scale from 0 to 60 [24]. We excluded one of the items that asked about whether the subject experienced lack of hunger, to avoid artificial associations with calorie intake induced by the construction of the scale; therefore, our resulting scale ranged from 0 to 57 instead of 0 to 60. In the original scale, subjects with a score of 16 or more points are typically classified as having MDD, which is also the cutoff we used for our study [24].

#### Physical activity

PA level among participants was measured through a self-administered questionnaire that has been used in similar follow-up studies [28]. This questionnaire was validated in its Spanish-translated version, and has been adapted to be used in Mexican populations [29].

The questionnaire estimates leisure time of PA in minutes per week during a typical week within the past year. The activities listed include walking, running, cycling, aerobics, dancing, swimming, and playing football, volleyball, basketball, tennis, baseball, softball, and squash, among others. The PA questionnaire contains time expressed in intervals (minutes or hours) and PA intensity (light, moderate, vigorous). Time (hours per week) and intensity of each leisure time physical activity (LTPA) was processed according to the Compendium of physical activities [30] to calculate metabolic equivalents (MET). Finally, weekly energy expenditure, was expressed in MET-hours per week.

#### Body fat

Participants had the body composition evaluation in fasting conditions and without practicing exercise the day before the evaluation. Body fat mass was determined by Dual-energy X-ray Absorptiometry (DEXA, Lunar DPX-GE, Lunar Radiation; software version 1.35, fast scan mode). The procedure for the DEXA consists of a subject being scanned with dual energy photons, whereby the scanner differentiates bones, fat free mass, and total fat mass of the body. For this study, the same technician, specifically trained to handle the equipment, performed all scans. The DEXA was calibrated with a phantom as indicated by standard procedures [31] and provided the absolute amount of body fat mass (kg) that was used for the present analysis.

#### Calorie intake

A semi-quantitative Food Frequency Questionnaire (FFQ), previously validated in Mexican populations [32] was used to assess dietary intake from the previous year and to get calorie intake estimation. The FFQ included data regarding the consumption of 116 food items. For each food, a commonly used portion size was specified (e.g., 1 slice of bread or 1 cup of coffee), and participants reported the frequency with which they had consumed each specific food during the previous year. Response options were 1 of 10 mutually exclusive possibilities, ranging from “never” to “6 or more times per day”. The frequency of each food was converted into daily intake and total energy intake was computed by summing the energy intakes from all foods.

#### Ethical considerations

The National Research Commission and Ethics Committee from the Social Security Mexican Institute (*Instituto Mexicano del Seguro Social*) evaluated and approved all the study procedures. The participation in the study was voluntary, and all participants signed the informed consent form.

### Statistical analysis

We conducted descriptive statistical analysis of the main variables of interest at baseline and follow-up, using central tendency (mean, median) and dispersion (standard deviation, interquartile interval) measures.

#### Model specification

**Fig. 1:**
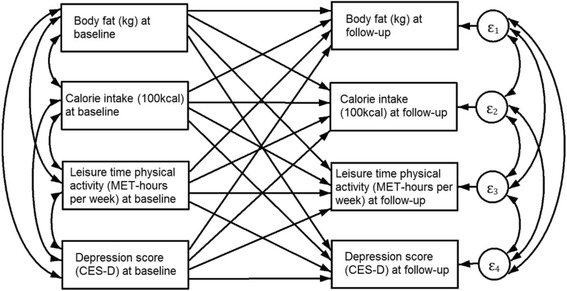
Cross-lagged Structural Equations Model with Depression Score, Body Fat, Calorie Intake and Leisure Time Physical activity at follow-up explained by their baseline levels and age at follow-up. Age at follow-up was included as explanatory variable in all equations (not shown in diagram for simplicity)

We used three SEM specifications and a hybrid mixed-effects model for each outcome. The latter approach allows the simultaneous estimation of fixed-effects coefficients (within subject or longitudinal relationship) and between-subject associations [33]. The first SEM was a cross-lagged model (Fig. 1). In this type of model, a group of variables at follow-up are used as endogenous (outcome variables), and the lagged version of these variables are specified as exogenous in each equation. The outcome variables for our analysis were body fat, calorie intake, LTPA and depression, all measured at follow-up. In each of the four equations, baseline measurements of body fat, calorie intake, LTPA and depression were specified as exogenous variables along with age at follow-up. All covariance parameters between equation errors were specified as well as all co-variances between exogenous variables. Since the baseline version of the outcome was adjusted for in each equation, the rest of the path coefficients can be interpreted in terms of their relationship to a regressed change in the outcome (e.g., how a unit difference in the baseline depression score relates to regressed changes in calorie intake). Although this type of SEM is common for longitudinal data, it does not completely isolate the within-subject relationship. In order to estimate both within and between subject relationships, we specified a hybrid mixed-effects model for each outcome; this type of model reproduces the coefficients from a fixed-effects model with the advantage of estimating between-subject relationships in the same model. For example, for body fat the model was specified as in the following equation

$$ {\displaystyle \begin{array}{l}{BF}_{it}={\beta}_0+{\beta}_1\left({calories}_{it}-{\overline{calories}}_i\right)+{\beta}_2{\overline{calories}}_i+{\beta}_3\left({LTPA}_{it}-{\overline{LTPA}}_i\right)+{\beta}_4{\overline{LTPA}}_i\\ {}+{\beta}_5\left({depression}_{it}-{\overline{depression}}_i\right)+{\beta}_6{\overline{depression}}_i+{b}_i+{e}_{it}\end{array}} $$

Where *i* = 1,  .. , *n* and *t* = 0, 1. The within or longitudinal relationship between calories and body fat is estimated by the parameter *β*_1_ and its point estimate corresponds to what would be obtained in a fixed-effects model whereas the between subject relationship between calories and body fat is estimated by the parameter*β*_2_, the coefficients of the other explanatory variables follows the same type of interpretation. In this model specification, ${b}_i\sim Normal\left(0,{\sigma}_b^2\right)$ is a random effect at the level of subjects assumed to be independent of the error ${e}_{it}\sim Normal\left(0,{\sigma}_e^2\right)$, both normally distributed with zero mean and constant variance. The correlation between measurements from the same subject is estimated by${\sigma}_b^2/\left({\sigma}_b^2+{\sigma}_e^2\right)$.

**Fig. 2:**
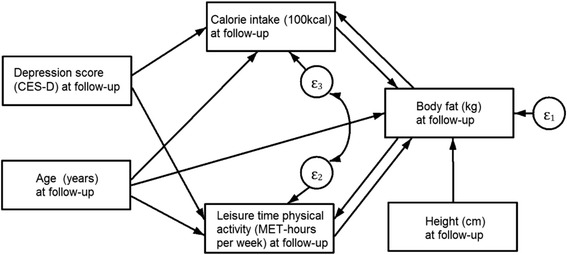
Structural Equation Model with Follow-up data with Calorie Intake and Leisure Time Physical Activity as mediators between Depression and Body Fat. All variances and covariances parameters between exogenous variables were included in the model (not shown in path diagram for simplicity)

Since there were about seven years of separation between baseline and follow-up measurements, it was expected path coefficients to be attenuated in the cross-lagged SEM. This model works best when the measurement lag is not much longer than the time lag between the cause and its effect [14], and this also applies when estimating the within-subject relation. When there are bi-directional or reciprocal relationships between variables but time lag of measurement is large, using a cross-sectional model can complement the cross-lagged model. We specified a second model using information from the follow-up exclusively (Fig. 2), which potentially has the advantage of recovering some of the relationships, but with the limitations of a cross-sectional model. Of special interest were the estimation of paths between health-related behaviors (diet, LTPA) and body fat and how these behaviors were explained by depression. We specified a set of three structural equations using body fat (kg) as the main outcome, and calorie intake and LTPA as mediator variables between depression and body fat (Fig. 2). We set a covariance parameter between LTPA and calorie intake equation errors to account for omitted variables that affect both mediators. We specified reciprocal relationships between body fat and calorie intake and between body fat and LTPA. Since our model was not recursive, instrumental variables had to be specified to achieve identification [34]. Instrumental variables are assumed to have a direct effect on the instrumented outcome but they are not related to the error in other equations where the instrumented variable functions as an explanatory variable. Height was assumed to instrument body fat, and we directly related depression only to calorie intake and LTPA. As an additional model, we added a path in the direction from body fat to depression to our cross-sectional specification.

**Fig. 3:**
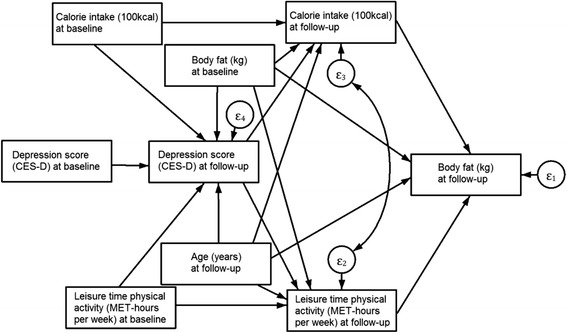
Structural Equation Model with Calorie Intake and Physical Activity as mediators between Depression and Body Fat at follow-up and with baseline covariates. All variances and covariances parameters between exogenous variables were included in the model (not shown in path diagram for simplicity)

Finally, we specified an intermediate model between the two specifications previously described (Fig. 3). We maintained the paths from health-related behaviors at follow-up to body fat at follow-up but instead of specifying contemporary reciprocity we used lagged fat mas as an explanatory variable for health-related behaviors at follow-up. We set up an additional equation for depression at follow-up with lagged health-related behaviors and lagged body fat as explanatory variables. All equations included the lagged outcome and age at follow-up as explanatory variables. We specified a covariance parameter between errors of calorie intake and LTPA equations, and all covariance parameters between exogenous variables.

#### Model estimation

We estimated all SEM specifications with the asymptotic distribution-free method which does not require normality in outcome distributions [35]. The hybrid mixed-effects model was estimated by maximum likelihood. All variables were centered at the mean in all SEMs, except for body fat at follow-up, so that the constant from the body fat equation estimates the mean of body fat at follow-up.

#### Goodness of fit

We compared the assumed structure to the data and calculated goodness of fit measures for SEMs 2 and 3 (SEM 1 had zero degrees of freedom and therefore represented a saturated model with perfect fit). We used the chi-square statistic, and fit indexes such as the Comparative Fit Index (CFI) and the Tucker-Lewis Index (TLI). We also calculated the Root Mean Square Error of Approximation (RMSEA) and obtained the probability that it was below the cutoff of 0.05. We tested Model 2 for stability conditions given its reciprocal relationships. We conducted all analyses in Stata v.14 [36]. The level of statistical significance was set at *α* = 0.0.

## Results

Table 1 shows the descriptive statistics for the analytical sample of adult women (*n* = 456). At baseline age ranged from 18 to 78 years with an average of 45.8 ± 13.2 (± SD) years, the average time between baseline and follow-up measurements was 7.2 ± 1.2 years. Mean body fat increased from 26.3 ± 8.0 kg at baseline to 28.4 ± 8.4 kg at follow-up. In relation to BMI, there was a right shift in the distribution between study stages. At baseline 37.7% of the sample were overweight and 18.0% were obese, at follow-up these percentages increased to 44.1% and 21.1%, respectively. At both study stages, median calorie intake was around 1800 kcal and mean LTPA around 12 METs/wk. At both study stages, the mean of the depression score was 11.6 and approximately 25% were classified as having MDD as indicated by the upper limit of the interquartile intervals.

**Table 1:** Descriptive statistics for variables used in the analysis of a sample of 460 Mexican women health workers

	Median (P25, P75)	Mean ± SD
Baseline		
Age, years	46.0 (37.0, 55.0)	45.8 ± 13.2
Body fat, kg	25.4 (20.7, 30.8)	26.3 ± 8.0
Weight, kg	61.5 (55.5, 69.5)	63.1 ± 11.5
Height, cm	155.0 (151.0, 159.0)	155.2 ± 5.8
BMI, kg/cm2	25.5 (23.1, 28.5)	26.2 ± 4.5
Calorie intake, 100 kcal	18.8 (14.4, 24.2)	20.5 ± 9.0
Leisure time physical activity, METs/wk	5.0 (0.9, 18.1)	12.5 ± 18.2
Depression score, CES-D*	9.0 (4.0, 17.0)	11.6 ± 9.9
Follow-up		
Age, years	53.0 (44.0, 62.0)	53.0 ± 13.1
Body fat, kg	27.3 (22.9, 33.1)	28.4 ± 8.4
Weight, kg	63.0 (56.8, 71.2)	64.8 ± 11.7
Height, cm	154.0 (151.0, 159.0)	154.8 ± 6.1
BMI, kg/cm2	26.5 (23.9, 29.3)	27.0 ± 4.5
Calorie intake, 100 kcal	17.1 (12.5, 22.5)	18.5 ± 8.8
Leisure time physical activity, METs/wk	3.9 (0.4, 15.3)	12.0 ± 18.6
Depression score, CES-D*	9.0 (4.0, 16.0)	11.6 ± 9.8

*n* = 456

*Scaled 0–57, excludes an item that asked about whether the subject experienced lack of hunger

Results from the cross-lagged SEM model are shown in Table 2. Baseline calorie intake and LTPA were not significantly related to body fat at follow-up. However, depression at baseline was related to a reduction in LTPA at follow-up and to an increase in calorie intake at follow-up; an increase of 10 points in the depression score at baseline was associated with an increase of 108 ± 38 (± standard error) kcal at follow-up. On the other hand, baseline calorie intake was related to higher levels of depression at follow-up. There was no statistically significant association from body fat at baseline to depression at follow-up, nor in the opposite direction (from baseline depression to follow-up body fat). Results from the hybrid mixed-effects model showed no statistically significant within-subject associations (Table 3). Regarding between-subject associations, depression and calorie intake were significantly related (*p* < 0.001). Results suggested an association (*p* = 0.098) between depression and body fat.

**Table 2:** Cross-lagged Structural Equations Model with Depression Score, Body Fat, Calorie Intake and Leisure Time Physical activity at follow-up explained by their baseline levels and age at follow-up

Explanatory variable	Coefficient ± Standard Error	*P*
Body fat at follow-up Equation, kg		
Depression score at baseline, CES-D	0.007 ± 0.021	0.725
Leisure time physical activity at baseline, METs/wk	−0.017 ± 0.013	0.177
Calorie Intake at baseline, 100 kcal	0.020 ± 0.025	0.435
Body fat at baseline, kg	0.924 ± 0.032	< 0.001
Age at follow-up	−0.111 ± 0.017	< 0.001
Constant	28.455 ± 0.197	< 0.001
Leisure time physical activity at follow-up equation, METs/wk		
Depression score at baseline, CES-D	−0.140 ± 0.063	0.026
Leisure time physical activity at baseline, METs/wk	0.260 ± 0.062	< 0.001
Calorie Intake at baseline, 100 kcal	0.109 ± 0.091	0.230
Body fat at baseline, kg	0.027 ± 0.098	0.781
Age at follow-up	0.036 ± 0.059	0.543
Constant	12.211 ± 0.858	< 0.001
Calorie Intake at follow-up, 100 kcal		
Depression score at baseline, CES-D	0.108 ± 0.038	0.005
Leisure time physical activity at baseline, METs/wk	−0.018 ± 0.021	0.388
Calorie Intake at baseline, 100 kcal	0.343 ± 0.063	< 0.001
Body fat at baseline, kg	−0.029 ± 0.056	0.611
Age at follow-up	−0.057 ± 0.031	0.066
Constant	18.822 ± 0.386	< 0.001
Depression score at follow-up, CES-D*		
Depression score at baseline, CESD	0.439 ± 0.046	< 0.001
Leisure time physical activity at baseline, METs/wk	−0.018 ± 0.022	0.412
Calorie Intake at baseline, 100 kcal	0.084 ± 0.042	0.046
Body fat at baseline, kg	0.073 ± 0.051	0.151
Age at follow-up	−0.052 ± 0.033	0.116
Constant	11.650 ± 0.402	< 0.001
Error Covariances		
Body fat with physical activity	−1.532 ± 3.628	0.673
Body fat with calorie intake	2.492 ± 1.471	0.090
Body fat with depression score	1.268 ± 1.839	0.491
Physical activity with calorie intake	−2.716 ± 5.687	0.633
Physical activity with depression score	−2.953 ± 7.124	0.678
Calorie intake with depression	7.824 ± 4.194	0.062

*n* = 456

Model estimated with the asymptotic distribution free method. All covariance parameters between exogenous variables were also included (available as Additional file 1: Table S1). All variables were centered at the mean except body fat at follow-up

*Scaled 0–57, excludes an item that asked about whether the subject experienced lack of hunger

**Table 3:** Mixed effects hybrid models for estimating within and between subject associations for each outcome variable

	Body Fat, kg		Calorie Intake, 100 kcal		Physical Activity, METs/week		Depression score, CES-D*	
	Coef ± SE	*P*	Coef ± SE	*P*	Coef ± SE	*P*	Coef ± SE	P
Within subject associations								
Depression score, CES-D	0.01 ± 0.023	0.667	−0.012 ± 0.047	0.790	0.068 ± 0.103	0.509		
Leisure time physical activity, METs/wk	−0.005 ± 0.01	0.616	−0.002 ± 0.021	0.928			0.014 ± 0.021	0.509
Calorie Intake, 100 kcal	−0.015 ± 0.023	0.514			−0.009 ± 0.103	0.928	−0.012 ± 0.047	0.790
Body fat, kg			−0.063 ± 0.096	0.514	−0.106 ± 0.211	0.616	0.041 ± 0.096	0.667
Between subject associations								
Depression score, CES-D	0.074 ± 0.044	0.098	**0.141 ± 0.04**	**<0.001**	−0.124 ± 0.082	0.131		
Leisure time physical activity, METs/wk	−0.016 ± 0.025	0.536	0.027 ± 0.023	0.250			−0.04 ± 0.026	0.131
Calorie Intake, 100 kcal	−0.02 ± 0.051	0.700			0.108 ± 0.094	0.250	**0.185 ± 0.053**	**<0.001**
Body fat, kg			−0.017 ± 0.043	0.700	−0.054 ± 0.087	0.536	0.081 ± 0.049	0.098
Constant	27.05 ± 1.143	<0.001	17.98 ± 1.324	<0.001	13.07 ± 3.103	<0.001	6.28 ± 1.77	<0.001
Correlation between measurements	0.815		0.331		0.251		0.442	

456 subjects; 2 measurements within subjects

*Scaled 0–57, excludes an item that asked about whether the subject experienced lack of hunger

Results from Model 2 (Fig. 2) that express the relationship from depression to body fat and the mediating role of health-related behaviors in this relationship using cross-sectional data are shown in Table 4. There was a positive and significant relationship from calorie intake to body fat; on average body fat was 328 ± 142 g higher for every increase of 100 kcal. On the other hand, LTPA was negatively and significantly related with body fat; for each MET/wk. increase of LTPA, on average, there was a decrease of 215 ± 91 g in body fat. Depression was positively associated with calorie intake; a unit increase in the depression score was related with an increase of about 19 ± 5 kcal, or equivalently, an increase of 184 ± 47 kcal for each standard deviation increase in the depression score (about 10 points). Depression was negatively related with METs of LTPA; for each increase in the depression score, METs from LTPA decreased on average 0.191 ± 0.095. That is, for an increase of one standard deviation in the depression score, METs from LTPA changed −1.87 ± 0.93. Once reciprocal effects and all indirect paths were accounted for and added to the direct effect of calorie intake on body fat, the relationship remained positive; an increase of 100 kcal was related with an increase of 245 g of body fat (±91, *p* = 0.007). The path coefficient from body fat to LTPA was statistically significant and with an opposite sign to the relation from LTPA to body fat. When indirect effects (including reciprocal ones) were added to the direct effect from LTPA to body fat, the total effect remained with the same sign as the direct effect: a unit increase in METs/wk. of LTPA was related to a total decrease of 161 (± 47, *p* = 0.001) grams in body fat.

**Table 4:** Structural Equation Model with Follow-up data with Calorie Intake and Leisure Time Physical Activity as mediators between Depression and Body Fat

Explanatory variable	Coefficient ± Standar Error	*P*
Body Fat at follow-up equation, kg		
Leisure time physical activity at follow-up, METs/wk	−0.215 ± 0.091	0.019
Calorie Intake at follow-up, 100 kcal	0.328 ± 0.142	0.021
Age at follow-up	0.115 ± 0.036	0.001
Height at follow-up	0.520 ± 0.092	< 0.001
Constant	28.399 ± 0.425	< 0.001
Leisure time physical activity at follow-up equation, METs/wk		
Body fat at follow-up, kg	1.059 ± 0.456	0.020
Depression score at follow-up, CES-D*	−0.191 ± 0.095	0.045
Age at follow-up	−0.025 ± 0.071	0.722
Constant	−30.038 ± 12.750	0.018
Calorie Intake at follow-up equation, 100 kcal		
Body fat at follow-up, kg	−0.341 ± 0.183	0.063
Depression score at follow-up, CES-D*	0.188 ± 0.048	< 0.001
Age at follow-up	−0.067 ± 0.035	0.057
Constant	9.630 ± 5.285	0.068
Error Covariances		
Calorie intake with physical activity	−26.882 ± 20.302	0.185

*n* = 456

Fit statistics: *χ*_1_=0.05, p = 0.830; RMSEA < 0.001, P(RMSEA < 0.05) =0.903; CFI = 1.0; TLI = 1.0

The model satisfied stability with all eigenvalues inside the unit circle

Model estimated with the asymptotic distribution free method. All covariance parameters between exogenous variables were also included (available as Additional file 1: Table S2). All variables were centered at the mean except body fat at follow-up

*Excludes an item that asked about whether the subject experienced lack of hunger

**Table 5:** Structural Equation Model with Calorie Intake and Physical Activity as mediators between Depression and Body Fat and with baseline covariates

Explanatory variable	Coefficient ± Standard Error	*P*
Body Fat at follow-up Equation, kg		
Leisure time physical activity at follow-up, METs/wk	−0.004 ± 0.011	0.700
Calorie Intake at follow-up 100 kcal	0.039 ± 0.023	0.099
Body fat at baseline, kg	0.931 ± 0.030	< 0.001
Age at follow-up	−0.102 ± 0.017	< 0.001
Constant	28.459 ± 0.196	< 0.001
Leisure time physical activity at follow-up equation, METs/wk		
Depression score at follow-up, CESD	−0.152 ± 0.074	0.041
Leisure time physical activity at baseline, METs/wk	0.262 ± 0.058	0.000
Body fat at baseline, kg	0.020 ± 0.098	0.839
Age at follow-up	0.030 ± 0.058	0.608
Constant	−0.261 ± 0.824	0.752
Calorie Intake at follow-up, 100 kcal		
Depression score at follow-up, CESD	0.151 ± 0.044	0.001
Calorie Intake at baseline, 100 kcal	0.316 ± 0.065	0.000
Body fat at baseline, kg	−0.027 ± 0.056	0.636
Age at follow-up	−0.042 ± 0.030	0.162
Constant	0.130 ± 0.374	0.729
Depression score at follow-up, CES-D*		
Leisure time physical activity at baseline, METs/wk	−0.020 ± 0.020	0.324
Calorie Intake at baseline, 100 kcal	0.078 ± 0.042	0.062
Depression score at baseline, CESD	0.455 ± 0.046	0.000
Body fat at baseline, kg	0.076 ± 0.050	0.126
Age at follow-up	−0.059 ± 0.033	0.072
Constant	0.030 ± 0.398	0.940
Error Covariances		
Calorie intake with physical activity	−2.371 ± 5.362	0.658

*n* = 456

Fit statistics: *χ*_8_=7.97, *p* = 0.437; RMSE < 0.001, P(RMSEA < 0.05) =0.921; CFI = 1.0; TLI = 1.0

Model estimated with the asymptotic distribution free method. All covariance parameters between exogenous variables were also included (available as Additional file 1: Table S2). All variables were centered at the mean except body fat at follow-up

*Excludes an item that asked about whether the subject experienced lack of hunger

Given that a higher depression score was related with a higher calorie intake and a lower level of LTPA, and a higher calorie intake and a lower level of LTPA were both related with higher kg of body fat, depression was found to be indirectly related to body fat. That is, a unit increase in the depression score was related with an increase of 77 (± 26, *p* = 0.004) grams of body fat through its effects on calorie intake and LTPA; this corresponds to an increase of more than half a kilo in body fat (751 ± 259 g) for each standard deviation increase in the depression score. Fit indices were adequate and the hypothesis that the model reproduced the data was not rejected (χ_1_ = 0.05, *p* = 0.830) (an additional analysis, we added a path from body fat to depression to Model 2, and the results were similar to those from Model 2, but the added path from body fat to depression was not statistically significant (*p* = 0.866, see Additional file 1: Table S3).

Results from Model 3 are shown in Table 5. There were no statistically significant paths from baseline body fat to health-related behaviors nor to depression at follow-up. Depression at follow-up was significantly related to a higher calorie intake and a lower LTPA at follow-up. Depression at baseline was also related to a higher calorie intake (0.069 ± 0.022, *p* = 0.001) and to lower LTPA (−0.069 ± 0.035, *p* = 0.047) through its effect of depression at follow-up. In this model without contemporary reciprocity, there were no statistically significant paths between health-related behaviors and body fat, although the path from calorie intake to body fat suggests a positive relationship (*p* = 0.099). Fit indices were adequate and the hypothesis that the model reproduced the data was not rejected$\left({\chi}_8^2=7.97,p=0.0437\right)$.

## Discussion

Using Structural Equations Models (SEM) methodology applied to a sample of female Mexican health workers, we estimated a cross-lagged model with depression, calorie intake, LTPA and body fat variables from two time points. To completely isolate within-subject associations we fitted a hybrid mixed-effects model to each outcome variable. We also fitted a cross-sectional SEM model where health-related behaviors (calorie intake and LTPA) acted as mediators between depression and body fat, and included reciprocal (bidirectional) relationships between body fat and health-related behaviors. Additionally, we specified an intermediate model that preserved the mediation structure but without reciprocity. Instead we included body fat at baseline as predictor in all equations.

Results from the cross-lagged model showed that higher depression at baseline was related to higher depression, higher dietary intake and lower LTPA at follow-up. On the other hand, higher baseline calorie intake was related to higher depression at follow-up. These results highlighted past depression as a common predictor for future health-related behaviors but also suggested that depression and dietary intake may respond to each other dynamically, reinforcing each other. We did not find a significant path from baseline LTPA to follow-up depression. Although there are studies indicating a relation from PA to depression [17, 37] it is also possible that a higher level of depression reduces PA [18].

In our cross-sectional model, we found that a higher depression score was related to a higher calorie intake and a lower LTPA, and these changes were in turn related to a higher body fat. Through its effects on calorie intake and LTPA, each standard deviation increase in the depression score was associated with an average increase of 751 ± 259 g (± standard error) in body fat. In a study among young US adults, Byedoun MA et al. included PA and a Health Eating Index (HEI) as mediators between MDD and BMI [37]. Consistent with our results, they found depression to be related to both PA and HEI, and PA significantly related to BMI. On the other hand, they did not find HEI significantly related to BMI among women. This discrepancy may be related to several factors, including the different definition of the dietary variable, measurement differences (24 h recall vs Food Frequency) or the type of model structure specified.

Although we found the total effects from health-related behaviors to body fat with the expected signs in our cross-sectional model, higher body fat was significantly related with higher LTPA and marginally related with lower calorie intake. These reverse relationships from body fat to health-related behaviors may be picking up a tendency of underreporting calorie intake and over-reporting LTPA among those with higher weight [38, 39].

However, we detected relationships between depression and health-related behaviors from our cross-lagged model using longitudinal data, no statistically significant paths were found in the body fat equation or from LTPA to depression. The lack of statistical significance for these relationships do not necessarily imply an absent relationship; study limitations including sample size and the time gap between measurements make it more difficult to detect such paths. For example, subjects could have felt periods of remission in depressive symptoms and variations in LTPA levels during the time gap between measurements. Furthermore, the hybrid mixed-effects models showed no statistically significant within-subject relationships between our key variables, this also indicates that time measurement separation may have hindered the detection of such relationships. On the other hand, if contemporary variables were used to estimate relationships using a cross-sectional approach, there would be no time gap between measurements but there would be the limitation of having only between-subject variation. We used a cross-sectional approach to test a proposed model structure in which health-related behaviors mediate between depression and body fat. A key aspect of our cross-sectional model was the inclusion of reciprocal relationships, and correlated errors between calorie intake and LTPA. Correlating errors from mediator equations is a way of taking into account the omitted variables that simultaneously affect these endogenous variables. Although we found significant relationships, it should be noted that these were based on between-subject data, results from the hybrid mixed-effects models also showed significant relationships but exclusively from between-subject associations. To better estimate within-subject (longitudinal) relationships, the study design is a key aspect that should be considered in future studies. Reciprocal relationships are best estimated with a carefully designed longitudinal study where the time gap between measurements does not exceed the time lag between a cause and an effect. When this is not the case a cross-sectional model could be useful [14, 40] to complement the analysis, but it would require the use of proper instrumental variables to tackle endogenous relationships among key study variables; this would ensure that the model is identified and estimators are efficient (less sampling variance). Given the complex relationships between variables, other paths could be added to our cross-sectional model specification given a bigger sample size and the availability of more instrumental variables.

Although we focused on the relationship in the direction from depression to body fat, it is also possible that body fat modifies depression. A cross-sectional study among Canadian females tested for reciprocity between BMI and depression and found a significant relationship from depression to BMI but not in the opposite direction [21]. Mannan et al., in a meta-analysis that included 19 prospective studies, reached the conclusion that there was a greater likelihood of depression leading to obesity than the converse; although the relationship from obesity to depression was statistically significant, it was stronger from depression to obesity [15]. We found no relationship in either direction between depression and body fat in our cross-lagged model. We also added a path from body fat to depression in our cross-sectional model but it was not statistically significant. Given the structure of our cross-sectional model, we found a higher depression score to be related to higher body fat through its effect on health-related behaviors.

To avoid or at least attenuate an induced relationship between dietary intake and depression, we excluded the item related to hunger from the depression scale. Regarding model estimation, we used the asymptotic distribution-free method that does not require normality in distributions of endogenous variables. Although we obtained good fit measures when our models were tested against the data, this does not imply that other model structures may also fit well.

From our cross-lagged effects model we observed that individuals with a higher baseline depression had a higher calorie intake and lower LTPA at follow-up. In contrast, within-subject associations from the hybrid mixed-effects model showed no significant associations. This indicates that there may be unobserved variables driving these associations between past depression and future health-related behaviors. Although these associations were detected even with a large time gap separation between measurements, future research is needed to explore the hidden mechanisms that may be driving the relationship between depression and health-related behaviors.

As was observed in this study, women with higher depression scores had, on average, a higher calorie intake and a lower LTPA. It is known that a calorie imbalance in turn generates an increase in body fat. These results are of interest to the health services sector, with implications in the design of programs and activities to reduce obesity and depression. The results also pointed out the importance in tackling challenging problems in public health, specifically how to effectively increase PA and prevent the rise of daily caloric intake. PA plays an important role in body weight and can promote long-term weight loss, especially when combined with reduced caloric intake [41]. It is also worth mentioning that PA has been considered as an alternative for prevention and treatment of the symptoms of depression [42, 43]. Interventions to prevent depression and promote PA among younger populations have particularly important implications, since the benefits are not only advantageous in the short-term but can also aid in preventing the future development of chronic diseases for which body weight is a risk factor, such as cancer and metabolic syndrome [44, 45]. However, the results of this study are not generalizable, as our study sample was not representative of the Mexican Population as a whole. This sample consisted of Mexican women who had IMSS medical insurance and better access to health care, not necessarily consistent with the general population of Mexico. This sample may be considered representative of seemingly healthy women from middle-income to low-income families residing in urban areas of the central region of Mexico.

## Conclusions

The results of this study showed that women with higher depression scores on average consumed more calories and reduced their LTPA, generating an energy imbalance that was reflected in a higher body fat. To the best of our knowledge this is the first study exploring these complex relationships in a Mexican population of adult woman and with body fat instead of indirect measures based on body weight and height. Our results are particularly relevant considering Mexico’s high prevalence of obesity combined with the effects that MDD might have on weight gain and other health outcomes associated with body weight excess such as diabetes cardiovascular disease. Evaluating the role of mental conditions like depression in dietary and PA behaviors should be positioned as a key research goal for better designed and targeted public health interventions.

## Additional file

Additional file 1: Table S1. Estimated error variances, variances of exogenous variables and covariances between exogenous variables from a Cross-lagged Structural Equations Model with Depression Score, Body Fat, Calorie Intake and Leisure Time Physical activity at follow-up explained by their baseline levels and age at follow-up. **Table S2.** Estimated error variances, variances of exogenous variables and covariances between exogenous variables from a Structural Equation Model with Follow-up data with Calorie Intake and Leisure Time Physical Activity as mediators between Depression and Body Fat. **Table S3.** Structural Equation Model with Follow-up data with Calorie Intake and Leisure Time Physical Activity as mediators between Depression and Body Fat, and with a direct path from Body Fat to Depression. (DOCX 21 kb)
