# Supplementary material for: Physical activity and calorie intake mediate the relationship from depression to body fat mass among female Mexican health workers
Source: Int J Behav Nutr Phys Act. 2017 Nov 17;14:160. doi: 10.1186/s12966-017-0612-x (PMC5693575; doi:10.1186/s12966-017-0612-x)
Supplement: Additional file 1: Table S1. — Estimated error variances, variances of exogenous variables and covariances between exogenous variables from a Cross-lagged Structural Equations Model with Depression Score, Body Fat, Calorie Intake and Leisure Time Physical activity at follow-up explained by their baseline levels and age at follow-up. Table S2. Estimated error variances, variances of exogenous variables and covariances between exogenous variables from a Structural Equation Model with Follow-up data with Calorie Intake and Leisure Time Physical Activity as mediators between Depression and Body Fat. Table S3. Structural Equation Model with Follow-up data with Calorie Intake and Leisure Time Physical Activity as mediators between Depression and Body Fat, and with a direct path from Body Fat to Depression. (DOCX 21 kb) [file 12966_2017_612_MOESM1_ESM.docx]

**Additional file 1**

**Table S1. Estimated error variances, variances of exogenous variables and covariances between exogenous variables from a Cross-lagged Structural Equations Model with Depression Score, Body Fat, Calorie Intake and Leisure Time Physical activity at follow-up explained by their baseline levels and age at follow-up**

|  | Parameter | Coefficient ± Standard Error | P |
| --- | --- | --- | --- |
| **Error variances** | | | |
|  | Body fat at follow-up | 17.634 ± 1.553 | < 0.001 |
|  | Leisure time physical activity at follow-up | 319.736 ± 49.501 | < 0.001 |
|  | Calorie Intake at follow-up | 64.851 ± 10.711 | < 0.001 |
|  | Depression score at follow-up | 73.910 ± 6.776 | < 0.001 |
| **Variances of exogenous variables** | | | |
|  | Body fat at baseline | 63.552 ± 5.369 | < 0.001 |
|  | Leisure time physical activity at baseline | 330.402 ± 49.302 | < 0.001 |
|  | Calorie Intake at baseline | 80.147 ± 8.654 | < 0.001 |
|  | Depression score at baseline | 97.694 ± 8.501 | < 0.001 |
|  | Age at follow-up | 171.616 ± 10.059 | < 0.001 |
| **Covariances between exogenous variables** | | | |
|  | Depression at baseline with LTPA at baseline | -2.558 ± 6.879 | 0.710 |
|  | Depression at baseline with Calorie Intake at baseline | 2.578 ± 3.788 | 0.496 |
|  | Depression at baseline with body fat at baseline | 3.061 ± 4.082 | 0.453 |
|  | Depression at baseline with Age at follow-up | -10.512 ± 7.277 | 0.149 |
|  | LTPA at baseline with Calorie Intake at baseline | 10.304 ± 6.760 | 0.127 |
|  | LTPA at baseline with body fat at baseline | -6.197 ± 6.801 | 0.362 |
|  | LTPA at baseline with age at follow-up | -32.987 ± 12.451 | 0.008 |
|  | Calorie intake at baseline with body fat at baseline | -1.438 ± 3.474 | 0.679 |
|  | Calorie intake at baseline with age at follow-up | -8.401 ± 6.350 | 0.186 |
|  | Body fat at baseline with Age at follow-up | 20.903 ± 4.643 | < 0.001 |

n=456

**Table S2. Estimated error variances, variances of exogenous variables and covariances between exogenous variables from a Structural Equation Model with Follow-up data with Calorie Intake and Leisure Time Physical Activity as mediators between Depression and Body Fat**

|  | Parameter | Coefficient ± Standard Error | P |
| --- | --- | --- | --- |
| **Error Variances** | |  |  |
|  | Body fat at follow-up, kg | 83.127 ± 15.158 | < 0.001 |
|  | Leisure time physical activity at follow-up, METs/wk | 425.289 ± 90.644 | < 0.001 |
|  | Calorie Intake at follow-up, 100kcal | 81.767 ± 16.816 | < 0.001 |
| **Variances of exogenous variables** | |  |  |
|  | Depression at follow-up | 95.080 ± 7.708 | < 0.001 |
|  | Age at follow-up | 171.617 ± 10.061 | < 0.001 |
|  | Height at follow-up | 37.488 ± 2.480 | < 0.001 |
| **Covariances between exogenous variables** | |  |  |
|  | Age at follow-up with Height at follow-up | -28.377 ± 4.102 | < 0.001 |
|  | Age at follow-up with Depression at follow-up | -12.183 ± 6.693 | 0.069 |
|  | Height at follow-up with Depression at follow-up | 1.827 ± 2.742 | 0.505 |

n=456

**Table S3. Structural Equation Model with Follow-up data with Calorie Intake and Leisure Time Physical Activity as mediators between Depression and Body Fat, and with a direct path from Body Fat to Depression**

|  | Explanatory Variable | Coefficient ± Error Estándar | P |
| --- | --- | --- | --- |
| **Body Fat at follow-up equation, kg** | | | |
|  | Leisure time physical activity at follow-up, METs/wk | -0.213 ± 0.092 | 0.020 |
|  | Calorie Intake at follow-up, 100kcal | 0.315 ± 0.159 | 0.048 |
|  | Age at follow-up | 0.113 ± 0.038 | 0.003 |
|  | Height at follow-up | 0.517 ± 0.092 | < 0.001 |
|  | Constant | 28.401 ± 0.423 | < 0.001 |
| **Leisure time physical activity at follow-up equation, METs/wk** | | | |
|  | Body fat at follow-up, kg | 1.047 ± 0.459 | 0.023 |
|  | Depression score at follow-up, CES-D | -0.182 ± 0.110 | 0.097 |
|  | Age at follow-up | -0.024 ± 0.071 | 0.739 |
|  | Constant | -29.690 ± 12.842 | 0.021 |
| **Calorie Intake at follow-up equation, 100kcal** | | | |
|  | Body fat at follow-up, kg | -0.328 ± 0.198 | 0.098 |
|  | Depression score at follow-up, CES-D | 0.183 ± 0.055 | 0.001 |
|  | Age at follow-up | -0.067 ± 0.035 | 0.057 |
|  | Constant | 9.258 ± 5.714 | 0.105 |
| **Depression at follow-up (CES-D)** | | | |
|  | Body fat at follow-up, kg | 0.012 ± 0.072 | 0.866 |
|  | Age at follow-up | -0.071 ± 0.039 | 0.065 |
|  | Constant | -0.323 ± 2.095 | 0.878 |
| **Error Covariances** | |  |  |
|  | Calorie intake with physical activity | -25.638 ± 21.186 | 0.226 |
| **Error Variances** | |  |  |
|  | Body fat at follow-up, kg | 82.351 ± 15.405 | < 0.001 |
|  | Leisure time physical activity at follow-up, METs/wk | 423.808 ± 90.293 | < 0.001 |
|  | Calorie Intake at follow-up, 100kcal | 81.196 ± 16.997 | < 0.001 |
|  | Depression score at follow-up, CES-D | 93.987 ± 7.606 | < 0.001 |
| **Variances of exogenous variables** | |  |  |
|  | Age at follow-up | 171.582 ± 10.057 | < 0.001 |
|  | Height at follow-up | 37.467 ± 2.481 | < 0.001 |
| **Covariances between exogenous variables** | |  |  |
|  | Age at follow-up with Height | -28.361 ± 4.104 | < 0.001 |

n=456

Fit statistics: $\chi_{1}$=0.02, p=0.878; RMSEA<0.001, P(RMSEA<0.05) =0.931; CFI=1.0; TLI=1.0

The model satisfied stability with all eigenvalues inside the unit circle. Model estimated with the asymptotic distribution free method. All variables were centered at the mean except body fat at follow-up
